# Effects of metformin on colorectal cancer stem cells depend on alterations in glutamine metabolism

**DOI:** 10.1038/s41598-017-18762-4

**Published:** 2018-01-11

**Authors:** Jae Hyun Kim, Kyoung Jin Lee, Yoojeong Seo, Ji-Hee Kwon, Jae Pil Yoon, Jo Yeon Kang, Hyun Jung Lee, Soo Jung Park, Sung Pil Hong, Jae Hee Cheon, Won Ho Kim, Tae Il Kim

**Affiliations:** 1 Department of Internal Medicine, Yonsei University College of Medicine, Seoul, Korea; 2 Institute of Gastroenterology, Yonsei University College of Medicine, Seoul, Korea; 3 Cancer Prevention Center, Yonsei University College of Medicine, Seoul, Korea; 4 Brain Korea 21 PLUS Project for Medical Science, Yonsei University, Seoul, Korea

## Abstract

Metformin has been known to suppress cancer stem cells (CSCs) in some cancers. However, the differential effects of metformin on CSCs and their mechanisms have not been reported. Herein, metformin induced pAMPK activation and pS6 suppression in metformin-sensitive (HT29) cells, but not in metformin-resistant (SW620) cells. The oxygen consumption rate was higher in HT29 cells than in SW620 cells and showed a prominent decrease after metformin treatment in HT29 cells. In glutamine-depleted medium, but not in low-glucose medium, SW620 cells became sensitive to the CSC-suppressing effect of metformin. A combination of metformin and glutaminase C inhibitor (compound 968) suppressed CSCs in SW620 cells and enhanced that effect in HT29 cells. SW620 cells showed higher expression of glutaminase 1 and glutamine transporter (ASCT2) than HT29 cells, especially ASCT2 in CSCs. Knockdown of glutaminase 1, ASCT2, and c-Myc induced significant CSC-suppression and enhanced CSC-suppressing effect of metformin and compound 968. In xenografts and human cancer organoids, combined treatment with metformin and compound 968 showed the same results as those shown *in vitro*. In conclusion, the effect of metformin on CSCs varies depending on the AMPK-mTOR and glutamine metabolism. The inhibition of glutamine pathway could enhance the CSC-suppressing effect of metformin, overcoming metformin resistance.

## Introduction

Colorectal cancer (CRC) is the fourth most common cancer-related cause of death in the world^1^. Despite recent developments in the treatment of CRC, the median overall survival time of patients with metastatic CRC is less than 30 months^2^. Cancers are heterogeneous, even within single tumors, as a consequence of genetic and epigenetic changes and microenvironmental differences^3^. Patients with CRC have varied responses to therapeutic agents, which might be caused by the different characteristics of each tumor, including MSI status, KRAS or BRAF mutation, CIMP status, and other unknown properties^4–6^. The optimal selection of therapeutic agents is important in the treatment of CRC. Cancer stem cells (CSCs) are considered one of the major causes limiting the treatment of cancers, including CRC^7,8^. The existence of CSCs within the bulk of the tumor might cause the tumor to escape from conventional chemotherapies, leading to relapse and metastasis^9^. Adjunctive treatment with CSC-suppressing agents could be a useful strategy to prevent recurrence and metastasis.

Metformin is considered a promising chemo-preventive agent and might have a suppressive effect on tumorigenesis and cancer-cell growth in several cancers. Recent studies suggest that metformin can inhibit cellular transformation and selectively suppress CSCs^10–12^. Some clinical studies showed that metformin reduced the risk of colorectal adenoma recurrence and cancer-specific mortality in patients with CRC and concurrent diabetes^13,14^.

Metformin is a classic biguanide drug used as a first-line therapy for type 2 diabetes. Among the many direct and indirect mechanisms for the anti-tumor effect of metformin, one of the primary targets of metformin is the respiratory chain complex I in the mitochondria. Cancer cells produce ATP and macromolecules for growth and proliferation by glycolysis and by mitochondrial metabolism including the TCA (tricarboxylic acid) cycle and oxidative phosphorylation (OXPHOS)^15^. Although glucose is the main energy source for most cancer cells, some cancer cells use glutamine for energy production, which is converted to α-ketoglutarate and entered into the TCA cycle^15^. Glutamine pathway is related with anabolic metabolism, predominantly producing macromolecules with less energy production. Recent studies have suggested that the inhibition of glutamine metabolism can potentiate the effects of metformin, reducing growth and inhibiting tumor progression^16,17^, in addition to showing that the downregulation of glutamine transporter (alanine, serine, cysteine-preferring transporter 2, ASCT2/SLC1A5) could suppress proliferation and induce apoptosis in CRC cell lines^18^. However, there have been no data on the interactive role of metformin-related effect and glutamine metabolic pathway on CSCs of CRC.

We show that metformin has different effects on CSCs of different CRC cell lines and hypothesize that the alternative glutamine pathway is related to the differential effects. We investigate the mechanism of the differential effects and evaluate the relevance of glucose and glutamine metabolism to the effects of metformin. We demonstrate a crucial role of the glutamine metabolic pathway in the development of resistance to the CSC-suppressing effects of metformin in CRC.

## Results

### Metformin had varied effects on CSCs of different CRC cell lines

**Figure 1:**
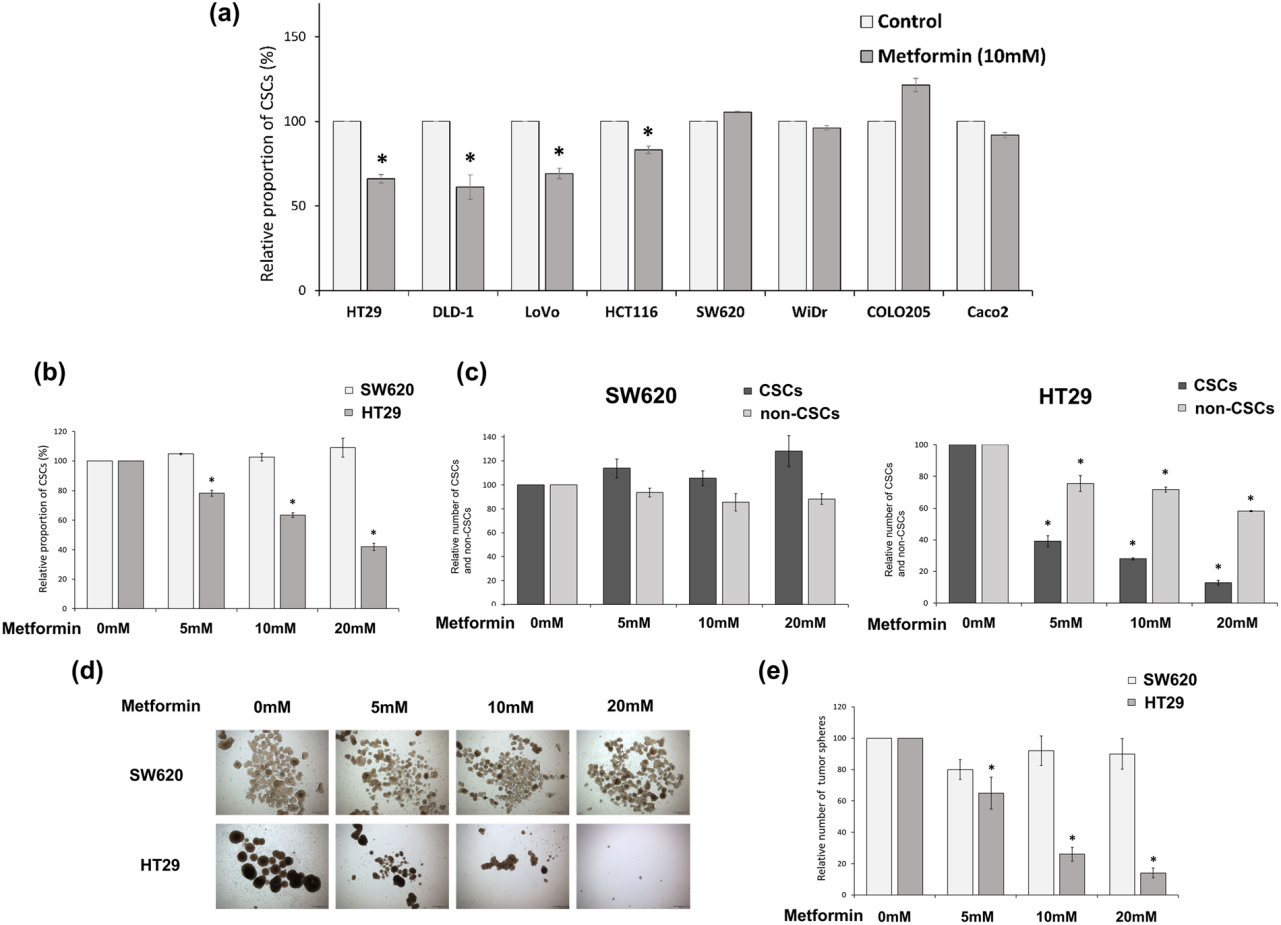
The different effects of metformin on CSCs of different CRC cell lines. (**a**) Metformin showed various CSC-suppressing effects in CRC cell lines. (**a** and **b**) The proportion of CD133^+^CD44^+^ cells (CSCs) was analyzed by flow cytometry using anti-CD133-PE and anti-CD44-FITC after 48 h treatment with control vehicle or metformin. (**c**) The numbers of CSCs and non-CSCs were counted and converted to proportions. (**d** and **e**) In tumor-sphere cultures with each concentration of metformin, the number of tumor spheres was counted at day 14 and compared to the counts in control cultures. Data are expressed as the mean ± standard error of three independent experiments; *P < 0.05 (compared with control).

We evaluated the effect of metformin on CSCs of each CRC cell line. We performed flow-cytometric analyses of CSC markers (CD133 and CD44) after treatment with metformin (10 mM). The proportion of CD133^+^CD44^+^ cells (CSCs) in four of the cell lines (HT29, DLD-1, LoVo, and HCT116) decreased significantly, whereas that in the other four cell lines (SW620, WiDR, COLO205, and Caco2) did not decrease (Fig. 1a). We selected two cell lines (HT29 [metformin-sensitive] and SW620 [metformin-resistant]) to investigate the mechanism of the differential effects of metformin. Flow cytometry showed that metformin significantly decreased the proportion of CSCs among the HT29 cells in a dose-dependent manner (control and 5 mM, 10 mM, and 20 mM metformin: 100% and 78.3%, 63.5%, and 41.9%, respectively; *P* < 0.001) but did not affect the proportion of CSCs among the SW620 cells (Fig. 1b). In addition, we counted and compared the CSCs and non-CSCs among the SW620 and HT29 cells (Fig. 1c). Among the SW620 cells, the proportions of CSCs and non-CSCs did not change significantly after the metformin treatment, whereas among the HT29 cells, both CSCs and non-CSCs decreased significantly after the metformin treatment, and the reduction of the CSCs was more pronounced than that of the non-CSCs. The number of tumor spheres significantly decreased after the metformin treatment in a dose-dependent manner in the HT29 cells (relative numbers of spheres in the control and 5 mM, 10 mM, and 20 mM metformin: 100% and 65.1%, 25.9%, and 13.9%, respectively; *P* < 0.001) but not in the SW620 cells (Fig. 1d and e). Thus, the metformin affected the CSCs differently in different CRC cell lines and affected CSCs more selectively than non-CSCs in the metformin-sensitive cell line.

### SW620 and HT29 cells showed different responses to AMPK activators and the same response to mTOR inhibitor in CSC-suppressing effects

**Figure 2:**
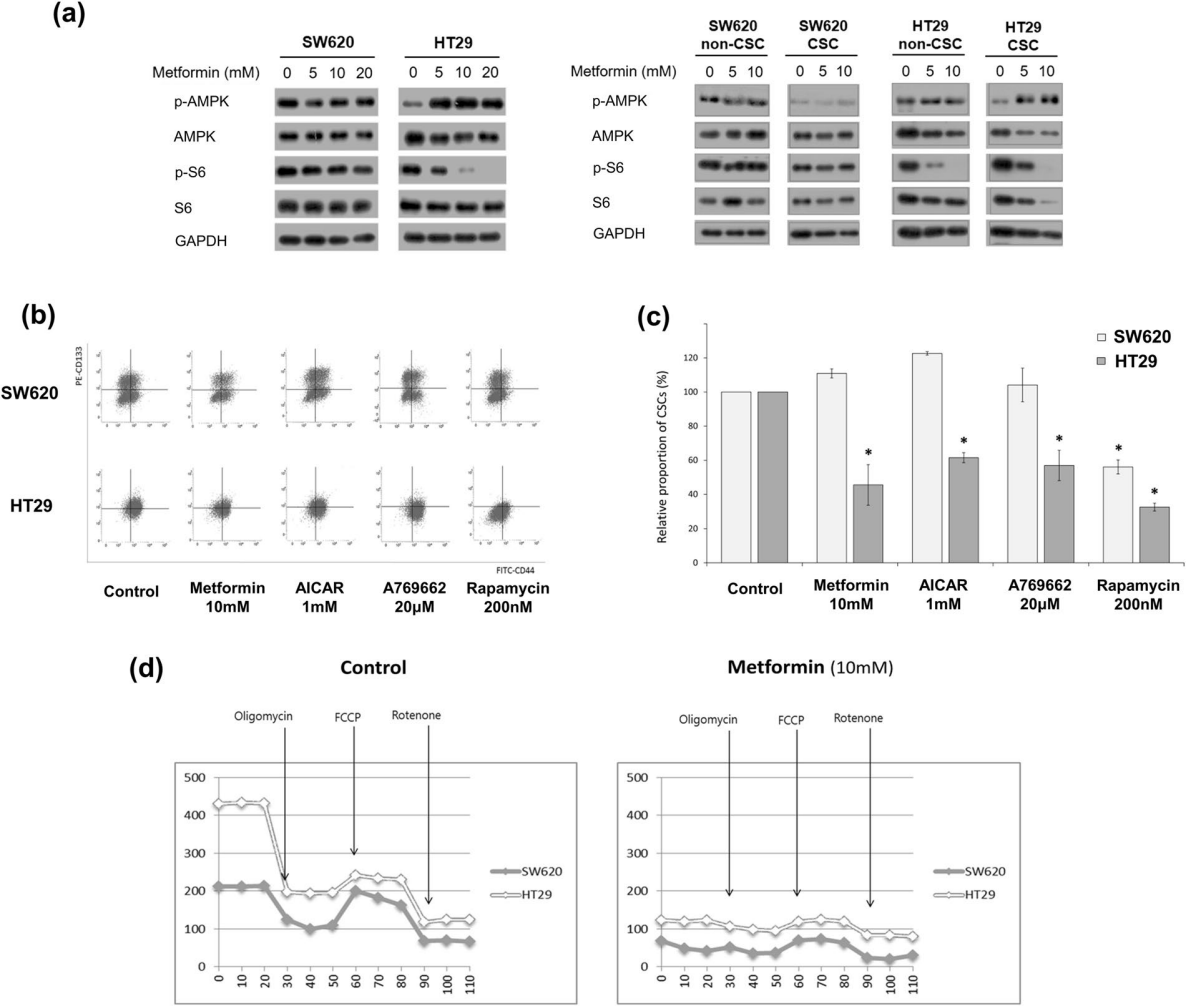
The different response of CSCs to AMPK/mTOR regulators and different dependence on oxidative phosphorylation between SW620 and HT29 cells. (**a**) In Western-blot analysis, the expression of phosphorylated S6 and phosphorylated AMPK in SW620 and HT29 cells was analyzed after 48 h treatment with control vehicle or metformin (5 mM, 10 mM, or 20 mM). CSCs (CD133^+^CD44^+^) and non-CSCs (CD133^−^CD44^−^) were sorted using FACS after the same treatment. (**b** and **c**) AICAR, A769662, and rapamycin were administered to evaluate the difference in responses to AMPK activators and mTOR inhibitor between SW620 cells and HT29 cells. The proportion of CD133^+^CD44^+^ cells in SW620 and HT29 cells was analyzed by flow cytometry after 48 h treatment with 10 mM metformin, 1 mM AICAR, 20 μM A769662, or 200 nM rapamycin. (**d**) The analysis of OXPHOS was performed using an XF (extracellular flux) analyzer after 48 h treatment with control vehicle or metformin (10 mM). Oligomycin (100 μM), FCCP (p-trifluoromethoxyphenylhydrazone, 100 μM), and rotenone (50 μM) were added, sequentially. Data are expressed as the mean ± standard error of three independent experiments; *P < 0.05 (compared with control).

To test whether the AMPK (adenosine monophosphate-activated protein kinase)-dependent mTOR (mammalian target of rapamycin) pathway, the most well-known mechanism of metformin action, is relevant to the difference in metformin-induced effects between SW620 and HT29 CSCs, we measured AMPK activation, mTOR suppression, and the change in the CSC proportion induced by metformin, AMPK activators, and rapamycin. In a Western-blot analysis, metformin induced pAMPK activation and pS6 suppression in HT29 cells but not in SW620 cells (Fig. 2a). We also performed Western-blot analysis using CSCs (CD133^+^CD44^+^) and non-CSCs (CD133^−^CD44^−^) sorted by FACS, and found similar results (Fig. 2a). In a flow-cytometric analysis, the proportion of CSCs was significantly decreased by AICAR (AMPK activator) or A769662 (direct AMPK activator) treatment in HT29 cells, as shown by metformin treatment; whereas the proportion of CSCs was not changed significantly by AICAR or A769662 treatment in SW620 cells. After treatment with rapamycin, the proportion of CSCs among both SW620 cells and HT29 cells decreased significantly (Fig. 2b and c). Those results demonstrate that both SW620 and HT29 CSCs are dependent on mTOR signaling; however, metformin-induced AMPK activation is involved in HT29 cells but not in SW620 cells.

### HT29 cells are more dependent than SW620 cells on oxidative phosphorylation

Metformin inhibits the respiratory chain complex I in the mitochondria and suppresses OXPHOS^19^. To investigate whether the energy-metabolism pathways are different between the two cell lines, we evaluated the OCR of SW620 and HT29 cells using an XF analyzer (Fig. 2d). In the assay, the OCR level of the HT29 cells was significantly higher than that of the SW620 cells. After metformin treatment, the OCR levels of both the SW620 cells and the HT29 cells decreased to the basal level, and the degree of reduction in the HT29 cells was significantly greater than that in the SW620 cells. Those results indicate that HT29 cells are more dependent than SW620 cells on OXPHOS for energy metabolism.

### The effects of low-glucose or glutamine-free conditions on CSCs of SW620 and HT29 cells

**Figure 3:**
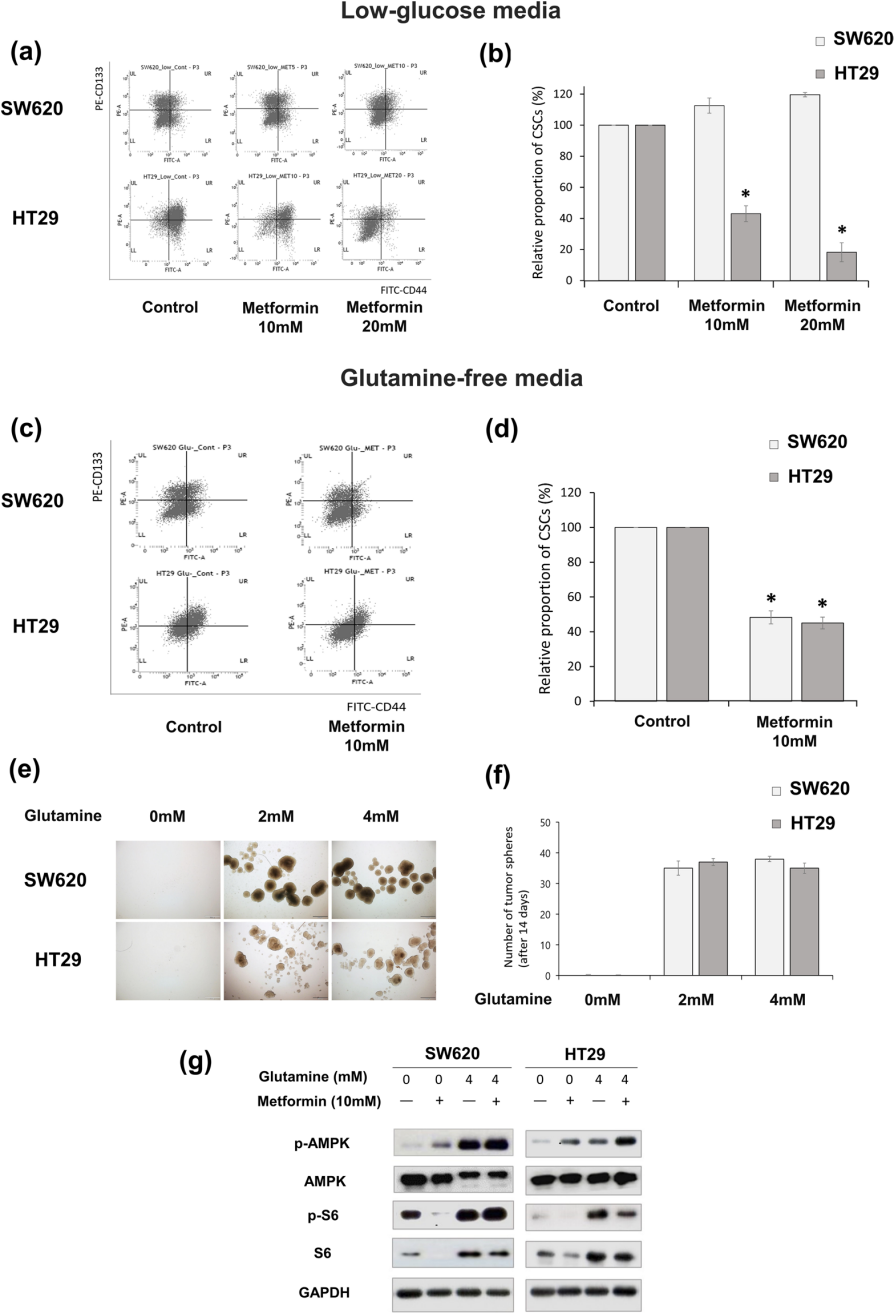
The different effects of low-glucose or glutamine-free conditions on CSCs of metformin-treated SW620 and HT29 cells. The control medium (Dulbecco’s modified Eagle’s medium) contained 4 mM L-glutamine and 4,500 mg/L glucose. (**a** and **b**) The low-glucose medium had the same composition except for 1,000 mg/L glucose. The proportion of CD133^+^CD44^+^ cells (CSCs) among SW620 and HT29 cells was analyzed by flow cytometry after 48 h treatment with control vehicle, 10 mM metformin, or 20 mM metformin. (**c** and **d**) The glutamine-free medium included DMEM with 10% FBS, 1% P/S, and 4,500 mg/L glucose. The proportion of CSCs among the SW620 cells and HT29 cells was analyzed by flow cytometry after 48 h treatment with control vehicle or 10 mM metformin. (**e** and **f**) In tumor-sphere culture, the number of colospheres was counted at day 14 in each concentration of glutamine. (**g**) In Western-blot analysis, the expression of p-S6/S6 and p-AMPK/AMPK in SW620 cells and HT29 cells was analyzed after 48 h treatment with control vehicle, L-glutamine, or metformin. Data are expressed as the mean ± standard error of three independent experiments; *P < 0.05 (compared with control).

Because glutamine can be an alternative energy source for tumor cells^15,20,21^, we tested the relative sensitivity of SW620 cells and HT29 cells to metformin in low-glucose or glutamine-free media. First, we investigated whether low-glucose or glutamine-free conditions alter the effects of metformin on SW620 and HT29 CSCs. In a low-glucose medium (DMEM/Low Glucose; 1000 mg/L glucose, HyClone, Logan, UT), the CSC-suppressing effect of metformin was enhanced in HT29 cells but not in SW620 cells (Fig. 3a and b). In a glutamine-free medium (DMEM without L-glutamine, HyClone, Logan, UT), the CSC-suppressing effect of metformin was induced in SW620 cells and enhanced in HT29 cells (Fig. 3c and d). In addition, tumor spheres of SW620 cells and HT29 cells could not survive in the glutamine-free medium (Fig. 3e and f). Those results reveal that glutamine is essential for the proliferation of SW620 and HT29 CSCs and indicate that HT29 cells are dependent on both glucose metabolism and glutamine metabolism, whereas SW620 cells are dependent on glutamine metabolism but not on glucose metabolism. This suggests that CSCs of SW620 cells show resistance to metformin due to glutamine-mediated compensation of energy source suppressed by metformin.

We also investigated whether the addition of glutamine and/or metformin affects AMPK-mTOR signaling in SW620 and HT29 cells. Western-blot analysis showed that the addition of glutamine induced activation of p-S6 in both SW620 and HT29 cells, and treatment of metformin induced activation of p-AMPK and suppression of p-S6 in HT29 cells, regardless of glutamine addition (Fig. 3g). However, in SW620 cells, treatment of metformin without glutamine induced activation of p-AMPK and suppression of p-S6, while treatment of metformin with glutamine addition did not (Fig. 3g). Those results reveal that the role of glutamine metabolism in the maintenance of CSCs via mTOR signaling, which is not affected by metformin treatment, is more essential in SW620 cells than in HT29 cells.

### The effect of inhibition of glutamine pathway on CSCs of SW620 and HT29 cells treated with/without metformin

**Figure 4:**
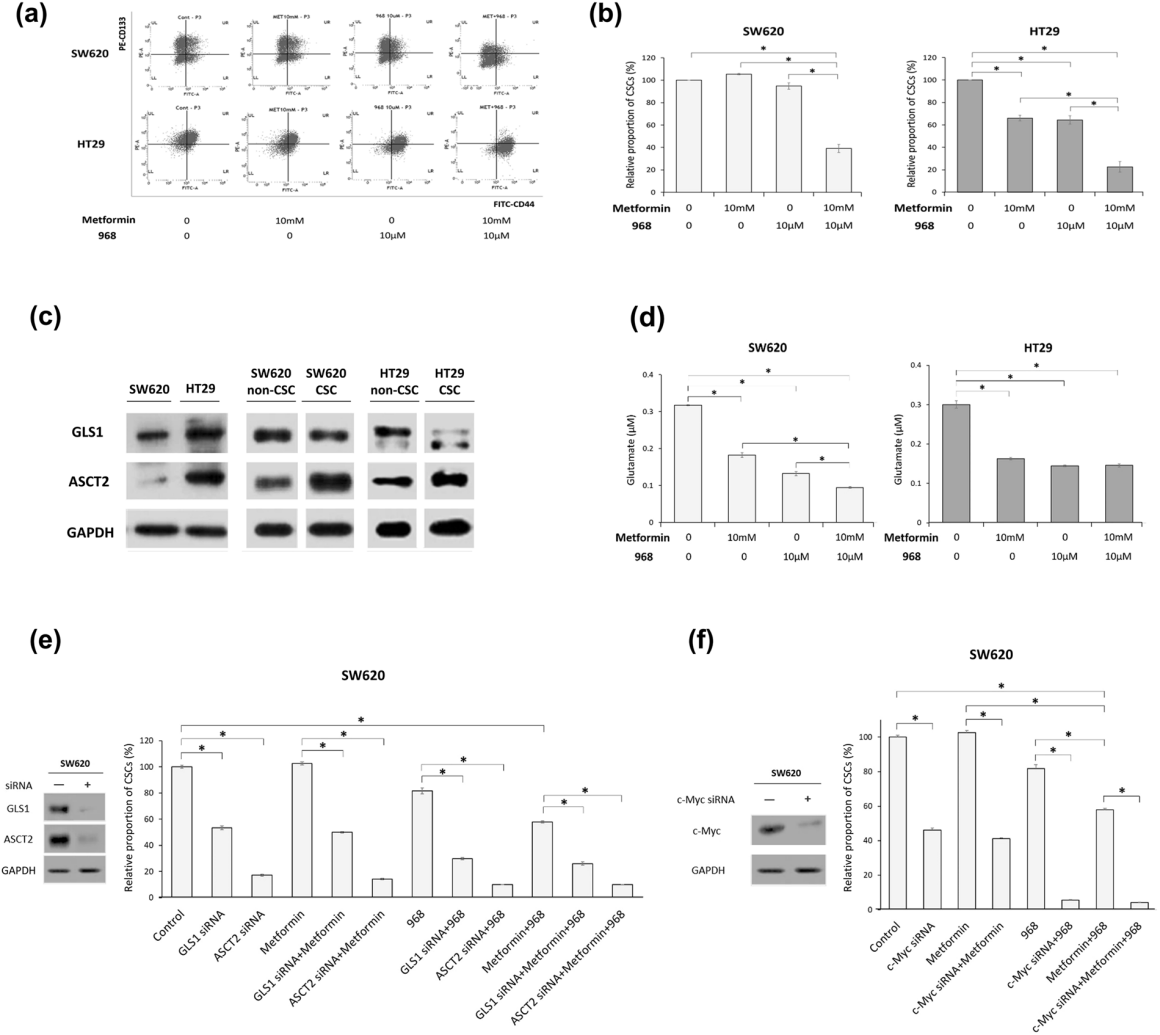
CSC-suppressing effects of metformin and/or inhibitors of glutamine metabolic pathway in CRC cell lines. (**a** and **b**) The inhibition of glutaminase induced the CSC-suppressing effect of metformin in SW620 cells and enhanced that effect in HT29 cells. (**a** and **b**) In SW620 cells and HT29 cells, the proportion of CD133^+^CD44^+^ cells (CSCs) was analyzed by flow cytometry after 48 h treatment with control vehicle, 10 mM metformin, 10 μM compound 968, or a combination of 10 mM metformin and 10 μM compound 968. (**c**) Protein expression of GLS1 and ASCT2 in SW620 cells and HT29 cells were evaluated by Western-blot analysis. CSCs (CD133^+^CD44^+^) and non-CSCs (CD133^−^CD44^−^) were sorted using FACS. Blot images of CSCs and non-CSCs were cropped from full image of the same experiment. Original image is provided in Supplementary Fig. S4. (**d**) In SW620 cells and HT29 cells, the amount of glutamate was analyzed by glutamate assay after the same treatment. (**e** and **f**) In knockdown experiments of GLS1, ASCT2 and c-Myc using siRNA in metformin-resistant SW620 cells, after transfection of each siRNA and 48 h treatment with metformin (10 mM) and/or compound 968 (10 μM), the proportion of CD133^+^CD44^+^ cells (CSCs) was analyzed by flow cytometry. The knockdown of each molecule was confirmed by Western-blot analysis. Data are expressed as the mean ± standard error of three independent experiments; *P < 0.05 (compared with control).

The anti-proliferative effect of metformin on cancer cells could be suppressed via increased glutamine metabolism, and combined treatment with glutaminase inhibitor and metformin might specifically enhance the beneficial effects of metformin in cancer treatment^16,17^. We evaluated whether the inhibition of glutaminase alters the effect of metformin on CSCs of SW620 and HT29 CSCs. In SW620 cells, treatment with either metformin or compound 968 (glutaminase C inhibitor) alone did not decrease the proportion of CSCs; however, combined treatment with metformin and compound 968 significantly decreased the proportion of CSCs (Fig. 4a and b). In HT29 cells, combined treatment with metformin and compound 968 enhanced the reduction in CSC proportions compared with the reductions caused by either metformin or compound 968 alone (Fig. 4a and b). Those results indicate that the combination of metformin and glutaminase inhibitor could induce a CSC-suppressing effect in metformin-resistant SW620 cells and that glutaminase inhibitor enhances that effect in metformin-sensitive HT29 cells.

Furthermore, the expression of GLS1 and ASCT2 in SW620 cells was higher than that in HT29 cells (Fig. 4c), suggesting that SW620 cells have more ability than HT29 cells to use glutamine as an energy source. We also found similar results in Western-blot analysis using CSCs (CD133^+^CD44^+^) and non-CSCs (CD133^−^CD44^−^) sorted by FACS (Fig. 4c). In particular, compared to non-CSCs, CSCs of both HT29 and SW620 cells showed higher expression of ASCT2 (Fig. 4c).

To confirm the effect of metformin and compound 968 on metabolic pathway, we performed glutamate assay under the same conditions. Then, we found that metformin treatment decreased the amount of glutamate, while combined treatment of metformin and compound 968 induced additive glutamate-decreasing effects in SW620 cells but not in HT29 cells, suggesting a greater contribution of glutamine metabolism in SW620 cells than in HT29 cells (Fig. 4d).

### Knockdown of GLS1, ASCT2, or c-Myc induced CSC-suppression, and enhanced CSC-suppressing effect of combining metformin and compound 968 in SW620 cells

To confirm the role of glutaminase 1 (GLS1) and ASCT2, the main determinants of glutamine metabolic pathway, in CSC maintenance via glutamine metabolism, we performed knockdown experiments of GLS1 and ASCT2 using siRNA in metformin resistant cell line SW620, and measured their effects on the proportion of CSCs. We found that knockdown of GLS1 or ASCT2 significantly decreased CSCs (CD133^+^CD44^+^) compared to the control (Fig. 4e). In addition, GLS1 knockdown enhanced the effects of glutaminase C inhibitor as well as the combination of metformin and glutaminase C inhibitor (Fig. 4e). CSC-suppressive effects of ASCT2 knockdown were more prominent than those of GLS1 knockdown (Fig. 4e), suggesting predominant dependency on glutamine metabolism in CSCs of SW620 cells. In addition, due to the higher expression of GLS1 and ASCT2 in CSCs of SW620 cells, particular with much more ASCT2 in CSCs, the potency of glutaminase C inhibitor (compound 968) in CSCs of SW620 may not be enough for suppression of glutamine metabolism, since the knockdown of GLS1 or ASCTs showed more significant CSC-suppression than glutaminase C inhibitor treatment, and enhanced the CSC-suppressive effect of glutaminase C inhibitor (Fig. 4e).

As c-Myc is known to be an important regulator of cancer metabolism, especially glutamine metabolism^22^, in examining the relationship between c-Myc and glutamine metabolism in our experiments, we performed Western-blot analysis for c-Myc, GLS1, and ASCT2 in eight CRC cell lines, and c-Myc knockdown experiments in SW620 cells. Western-blot analysis showed a relative correlation between c-Myc and GLS1/ASCT2 expression (Supplementary Fig. 1). Moreover, c-Myc knockdown induced significant CSC-suppression and enhanced CSC-suppressive effect of the combination of metformin and glutaminase C inhibitor in metformin-resistant SW620 cells (Fig. 4f).

### Various CSC-suppressing effects of metformin and/or glutaminase inhibitor in CRC cell lines

We assessed the effects of metformin and glutaminase C inhibitor on CSCs of the other six CRC cell lines (Supplementary Fig. 2). In the metformin-sensitive lines (DLD-1, LoVo, and HCT116), treatment with compound 968 alone significantly decreased the proportion of CSCs, as shown by treatment of metformin, and combined treatment with compound 968 and metformin showed various extents of enhancement of the CSC-suppressing effects of the two drugs, which were comparable to those in HT29 cells. In the metformin-resistant lines (WiDR, COLO205, and Caco2), treatment with compound 968 alone significantly decreased the proportion of CSCs, and combined treatment with compound 968 and metformin showed a minor enhancement effect. Those results suggest that the inhibition of glutamine metabolism contributes to the suppression of CSCs in metformin-resistant cell lines as well as in metformin-sensitive cell lines to various extents, based on the dependencies of the cells on major and alternative energy sources. Therefore, The CSCs of different cell lines might have varying abilities to use OXPHOS and/or glutamine metabolism, and to compensate for suppressed energy sources in different situations.

### Combined treatment with metformin and glutaminase inhibitor enhanced the effect of metformin and overcame the metformin resistance of CSCs in xenograft mice and in tumor organoids of human CRC

**Figure 5:**
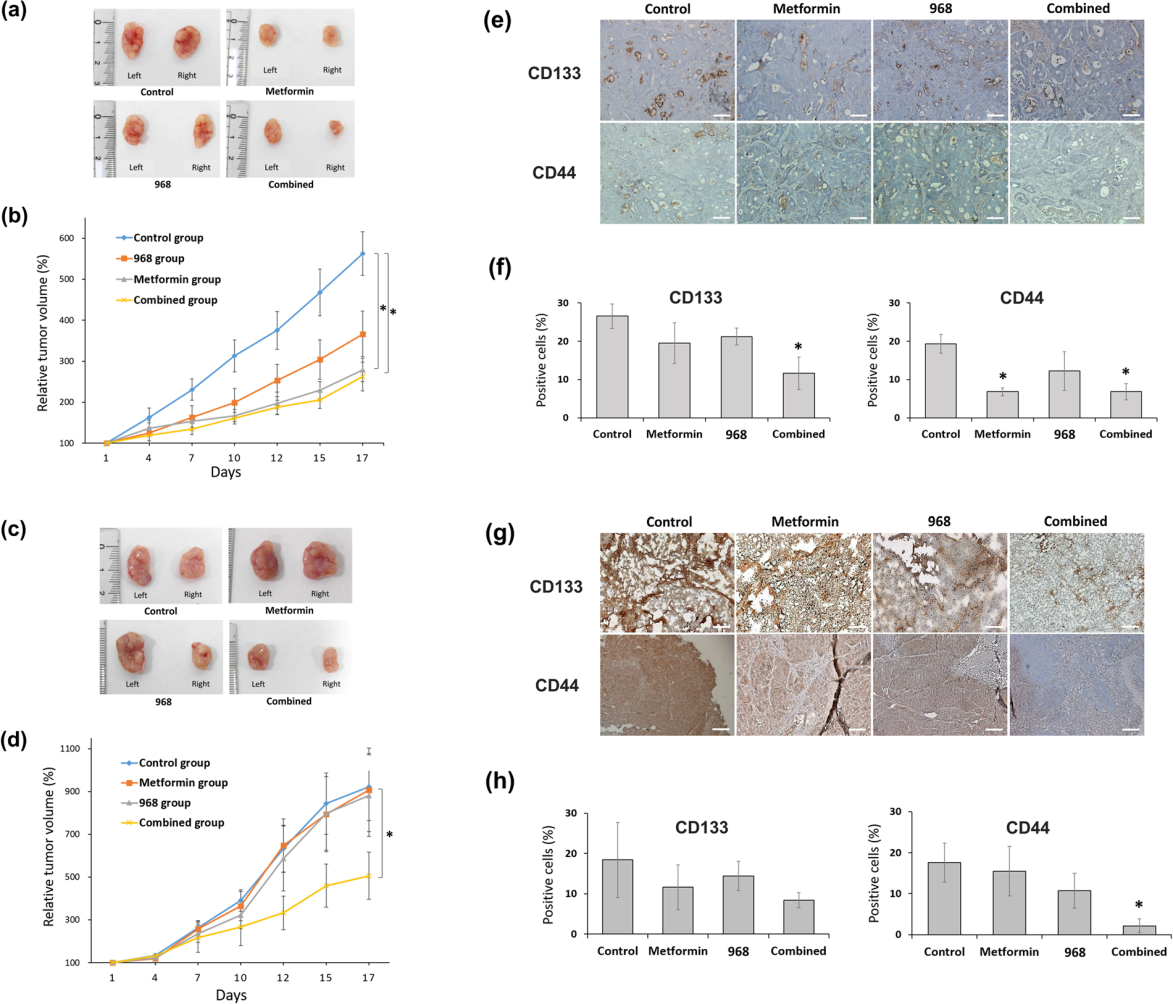
CSC-suppressing effects of metformin and/or glutaminase inhibitor in xenograft mice. SW620 cells or HT29 cells were injected subcutaneously into both flanks of nude mice. Twenty implanted mice were evenly allocated randomly into four groups: vehicle, metformin (by drinking water, 500 mg/kg/day), compound 968 (by intraperitoneal injection, 10 mg/kg with 10% DMSO in 200 μl PBS daily), and the combination of the same dose and frequency of metformin and compound 968. The size of the tumors was measured, and the mice were sacrificed at day 17. In the mice implanted with HT29 cells (**a** and **b**) and SW620 cells (**c** and **d**), the tumor growth of four groups was compared, respectively. Then, immunohistochemistry (IHC) of xenograft tumors treated with vehicle, metformin alone, compound 968 alone, and metformin combined with compound 968 was performed on frozen sections of tumor for CD133 and on sections of formalin-fixed, paraffin-embedded, dissected tumor samples for CD44. In the stained HT29 xenograft tumor (**e** and **f**) and SW620 xenograft tumor (**g** and **h**), the expression of CD44 and CD133 were evaluated. Graphs represent the percentage of CD133 or CD44 stained cells relative to all cells in the five different fields under x400 microscope. Data are expressed as the mean ± standard error; *P < 0.05 (compared with control). n = 5 per group, Scale bar = 100 μm.

In the mice implanted with HT29 cells (Fig. 5a and b), the tumor growth in the metformin and combined groups was suppressed by 50.5% (*P* = 0.003) and 53.2% (*P* = 0.007), respectively, relative to that in the control group at the end of study. The tumor growth in the 968 group was suppressed by 34.8% relative to that in the control group at day 17; however, the difference in tumor volume did not reach statistical significance (*P* = 0.210). In the mice implanted with SW620 cells (Fig. 5c and d), the tumor growth in the combined group was suppressed by 45.1% (*P* = 0.006) relative to that in the control group at day 17. However, the tumor volumes in the metformin group and the 968 group did not decrease significantly compared with that in the control group.

We performed IHC staining of xenograft tumors for CSC markers (CD133 and CD44). Quantitative assessment of the stained HT29 xenograft tumor revealed that treatment with metformin alone significantly decreased the proportion of CD44^+^ cells but not that of CD133^+^ cells (control group vs. metformin group: CD44^+^ cells, 19.3% vs. 6.8%, *P* < 0.001; CD133^+^ cells, 26.5% vs. 19.5%, *P* = 0.183), whereas treatment with compound 968 did not significantly decrease the proportion of cells positive for either marker (control group vs. 968 group: CD44^+^ cells, 19.3% vs. 12.2%, *P* = 0.152; CD133^+^ cells, 26.5% vs. 21.2%, *P* = 0.118). Combined treatment with metformin and glutaminase inhibitor significantly decreased the percentages of both CD133^+^ cells and CD44^+^ cells (control group vs. combined group: CD44^+^ cells, 19.3% vs. 2.8%, *P* < 0.001; CD133^+^ cells, 26.5% vs. 11.6%, *P* = 0.007; Fig. 5e and f). Quantitative analysis of the stained SW620 xenograft tumors revealed that metformin alone did not significantly reduce the percentages of CD133^+^ cells and CD44^+^ cells (control group vs. metformin group: CD44^+^ cells, 17.6% vs. 15.5%, *P* = 0.759; CD133^+^ cells, 18.4% vs. 11.6%, *P* = 0.493) and nor did compound 968 alone (control group vs. 968 group: CD44^+^ cells, 17.6% vs. 10.7%, *P* = 0.248; CD133^+^ cells, 18.4% vs. 14.4%, *P* = 0.662). However, combined treatment with metformin and glutaminase inhibitor significantly decreased the percentage of CD44^+^ cells but not that of CD133^+^ cells (control group vs. combined group: CD44^+^ cells, 17.6% vs. 2.1%, *P* = 0.008; CD133^+^ cells, 18.4% vs. 8.4%, *P* = 0.285; Fig. 5g and h).

**Figure 6:**
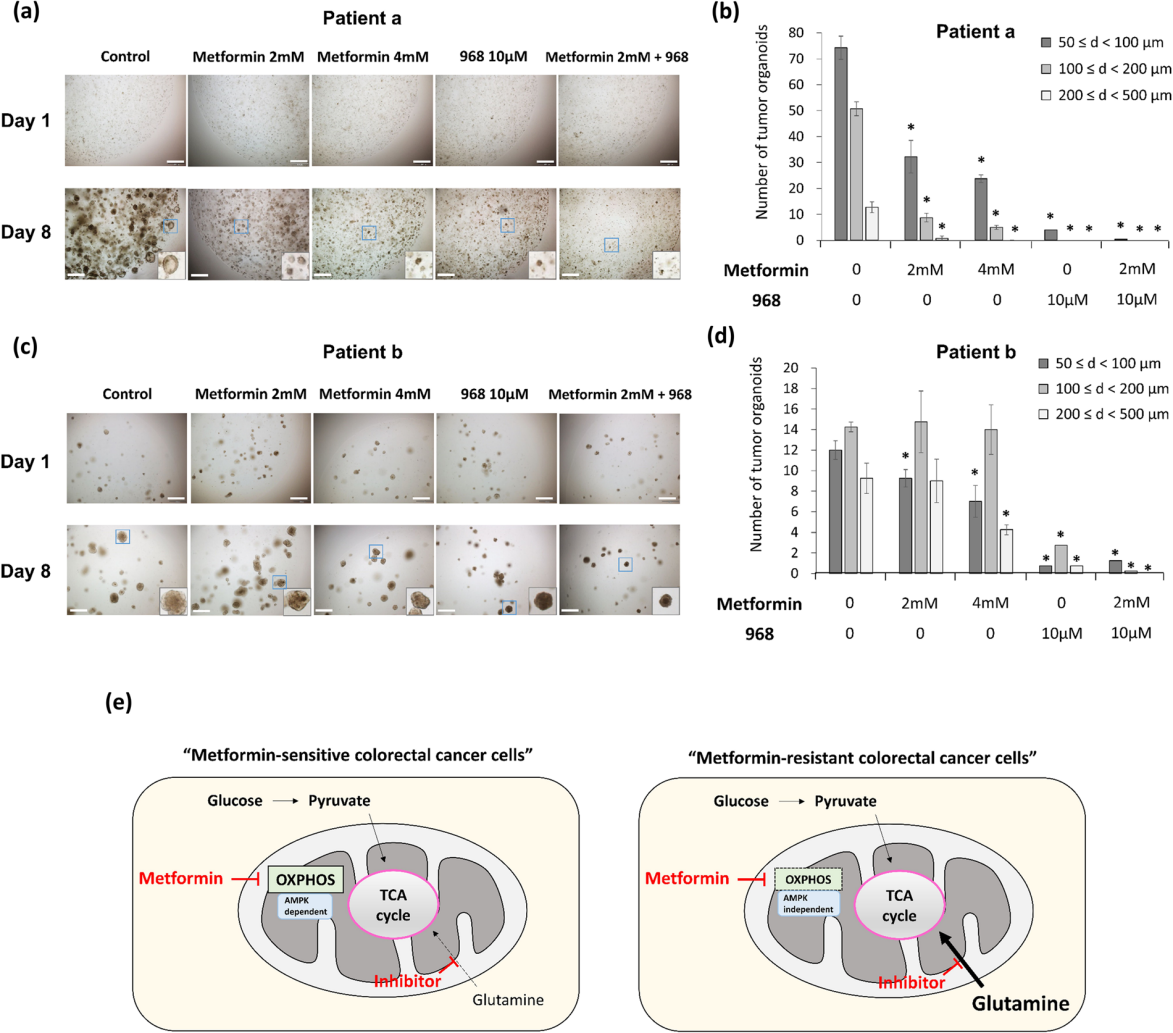
The effect of metformin and/or glutaminase inhibitor in human CRC organoids. Endoscopic biopsy samples were obtained from two patients with CRC. From the obtained tissues, tumor cells were isolated, embedded in Matrigel, and maintained in fresh basal culture medium for tumor organoid culture. Each medium was overlaid with control, metformin (2 mM or 4 mM), compound 968 (10 μM), and a combination of metformin (2 mM) and compound 968 (10 μM). The images of organoids were taken by a microscope (Olympus Bx51 microscope) at day 1 and day 8. The size and number of tumor organoids in patient a (**a** and **b**) and patient b (**c** and **d**) were measured, and tumor organoid counts within each size range (d, diameter of tumor organoid) were compared to counts for controls. (**e**) A model of the CSC-suppressing effect of metformin and glutamine metabolism inhibitor depending on the different metabolic determinants between the OXPHOS pathway and glutamine metabolism. The CSC-suppressing effect of metformin can be enhanced by inhibiting the contribution of glutamine to tumor metabolism. The contribution of glutamine metabolism in metformin-resistant cells is larger than that in metformin-sensitive cells, and the difference is dependent on the AMPK-mTOR pathway and the glutamine metabolic pathway. Consequently, the addition of glutamine inhibitor to metformin might be an effective strategy to enhance CSC-suppressing effect of metformin, overcoming the resistance to metformin. Data are expressed as the mean ± standard error of three independent experiments; *P < 0.05 (compared with control). Scale bar = 500 μm.

The same experiments using treatment with metformin and/or glutaminase inhibitor were performed with the tumor-organoid model of human CRC. Images of the organoids were taken using a microscope at day 1 and day 8. In cells from one patient (patient a), the size and number of tumor organoids decreased after treatment with metformin in a dose-dependent manner and also decreased after treatment with compound 968 alone. In addition, the combined treatment of those cells with metformin and compound 968 enhanced the effects on the size and number of tumor organoids (Fig. 6a and b). Those results were similar to the results of the experiments with the metformin-sensitive cell lines, including HT29 cells. In cells from the other patient (patient b), the size and number of tumor organoids did not change after treatment with metformin; however, treatment with compound 968 alone and combined treatment with metformin and compound 968 decreased the size and number of tumor organoids (Fig. 6c and d). Those results were similar to the results of the experiments with the metformin-resistant cell lines, including SW620 cells.

## Discussion

The effects of metformin on CSCs of each CRC cell line were different, and those differences depended on the AMPK-mTOR pathway and the glutamine metabolic pathway. In addition, the inhibition of glutamine metabolism pathway induced a CSC-suppressing effect of metformin in metformin-resistant cells and enhanced that effect in metformin-sensitive cells. To our knowledge, this is the first study to identify the differential effects of metformin on CSCs of different CRC cells as well as the mechanism of those differences, demonstrating an important role of glutamine metabolism.

Metformin has been identified as a chemo-preventive agent in CRC^9,23^, and studies in animal models showed that metformin induced AMPK activation and inhibited tumor development and growth, including colon tumorigenesis^24,25^. Metformin selectively killed CSCs in four genetically distinct types of breast cancer, and the combination of metformin and chemotherapeutic agent killed both CSCs and non-CSCs *in vitro* and *in vivo*^26^. In our experiments, metformin suppressed CSCs in some CRC cell lines, including HT29, but not in other CRC cell lines, including SW620. Because metformin transportation is one of the important determinants of the effects of metformin^19^, we measured the expression of metformin transporters in HT29 and SW620 cells. However, the expression of the metformin transporters, such as OCT1, OCT3, and MATE1, was not significantly different between the SW620 and HT29 cells (data not shown).

One of the major mechanisms of metformin effect is associated with the liver kinase B1-dependent activation of AMPK. The activated AMPK inhibits mTOR, which is a key regulator of growth factors as well as an important mediator of the PI3K/protein kinase B/Akt pathway in human cancer^19,27^. Our results showed that although both SW620 and HT29 CSCs were dependent on the regulation of mTOR signaling, metformin-induced AMPK activation was involved in metformin-sensitive HT29 cells but not in metformin-resistant SW620 cells. For the growth and proliferation of cancer cells, glycolysis and mitochondrial metabolism including the TCA cycle and OXPHOS play important roles in the production of ATP and macromolecules^15^. Metformin inhibits the respiratory chain complex I in the mitochondria, suppresses OXPHOS, and activates AMPK^19^. Song *et al*. showed that CSCs of CRC had higher OCR and mitochondrial ATP levels compared with non-CSCs of CRC^28^. We showed that HT29 cells use OXPHOS more than SW620 cells do. Those findings suggest that differences in cellular energy metabolism and AMPK pathway activation might be the cause of the different responses to metformin on CSCs among the CRC cell lines. Birsoy *et al*. showed that cancer cells exhibit diverse responses to glucose limitation and that the variable sensitivity to low glucose levels among cell lines is dependent on the upregulation or downregulation of OXPHOS^29^. Our results suggest that in the metformin-resistant cells, the alternative metabolism may contribute to the compensation for the limitation of glucose or suppression of OXPHOS by metformin.

Cancer cells differ from normal tissues in the characteristics of their energy metabolism, and they use glucose at a high rate for aerobic glycolysis^15^. In addition, cancer cells are extremely heterogeneous and have various metabolic phenotypes^30^. Increased aerobic glycolysis is a unique characteristic observed in cancers^31^. LDH-A (lactic dehydrogenase A), which catalyzes the transformation of pyruvate into lactate in the final step of the glycolytic pathway, plays a crucial role in glycolysis and is emerging as an important molecular target in cancer cells^32,33^. The inhibition of LDH-A might reduce the invasive and metastatic potential of cancer cells^34^. We investigated the effects of sodium oxamate, a classic inhibitor of LDH-A, with or without metformin on CSCs of CRC. The inhibition of LDH-A had few inhibitory effects on SW620 and HT29 CSCs under our experimental conditions (Supplementary Fig. 3a and b). Subsequently, we found that metabolic alteration via the glutamine pathway could play a critical role in the mechanism determining the differential effects of metformin on CSCs of CRC. Glutamine plays a key role as a metabolic fuel for intestinal epithelial proliferation and crypt expansion by activating intestinal stem cells via the mTOR pathway^35^. The role of the glutamine pathway could also be important for the proliferation and maintenance of CSCs of CRC. In some cancers, glutamine helps to compensate for glucose shortages^36–38^. We also found that the addition of a glutaminase inhibitor could induce the CSC-suppressing effect of metformin in metformin-resistant cells and enhance that effect in metformin-sensitive cells *in vitro, in vivo* in xenograft experiments, and in a human CRC-organoid model. Therefore, the role of glutamine metabolism in maintenance of CSCs via mTOR signaling is more essential in metformin-resistant SW620 cells than in metformin-sensitive HT29 cells. We suggest that glutamine metabolism plays a crucial role in the proliferation and maintenance of CSCs of CRCs, and that metformin and/or inhibitors of glutamine metabolism could help potentiate CSC-suppressing effects and decrease CRC recurrence and metastasis by inhibiting the role of CSCs.

As for glutamine metabolic pathway, in the presence of glutamine, CSCs of SW620 showed resistance to metformin treatment with no change of AMPK-mTOR pathway. However, without glutamine, they became sensitive to metformin treatment with activation of AMPK and suppression of mTOR, as HT29 cells did. Moreover, in major determinants of glutamine metabolic pathway, expressions of GLS1 and ASCT2 were higher in SW620 than in HT29 cells; and compared to non-CSCs, especially, CSCs of both cell lines showed higher expression of ASCT2. In addition, knockdown of ASCT2 induced much more significant suppression of CSCs in SW620 cells compared to both treatment of compound 968 and knockdown of GLS1, suggesting incomplete suppression of GLS due to low potency of compound 968 or remaining GLS2^39^. In this aspect, our results revealed that CSCs of SW620 had more prominent glutamine-dependent metabolism than those of HT-29 cells, and that potency of glutaminase C inhibitor was not enough to completely block glutamine pathway. Therefore, in the state of incomplete borderline block of glutamine metabolism in glutamine-dependent SW620 cells, combining metformin and glutaminase C inhibitor may show significant effect due to further blocking of the compensating metabolic pathway. This might explain the reason why SW620 cells showed CSC-suppressive effect in combined treatment of metformin and glutaminase inhibitor, and no effect when either metformin or glutaminase inhibitor was treated alone. Now, we need more complete blocking of glutamine metabolism pathway to suppress metformin-resistant and glutamine-dependent CSCs, especially by targeting ASCT2^40^ or both GLS1 and GLS2^39^.

In the future, it may be possible to apply more precise personalized treatment and prevention strategies for CRC based on biomarkers of tumor metabolism, which can identify major metabolic determinants of each tumor. However, considering the varying ability and flexibility to compensate for suppressed metabolic pathways and potency of metabolic inhibitor, as suggested by our experimental results, combined treatment targeting different metabolic pathways, like combined inhibition of OXPHOS and glutamine metabolic pathways, may provide more benefits regarding CSC-suppressing effect. In this aspect, we discovered CSC-specific glutamine-dependent metabolic difference and its contribution to metformin-resistance, which suggested usefulness of CSC-specific metabolic determinants like ASCT2 as biomarkers and targetable molecules for precise cancer treatment and new drug development.

In summary, the differential CSC-suppressing effects of metformin in CRC can be dependent on the relative regulation of the AMPK-mTOR pathway and the contribution of the glutamine metabolic pathway. The CSC-suppressive effect of metformin and/or inhibitor of glutamine metabolic pathway depends on the relative contribution of OXPHOS and glutamine metabolism, which differs between metformin-sensitive and -resistant CRC cells (Fig. 6e). Glutamine metabolism inhibitor and/or metformin could be an effective adjunctive treatment option to enhance the CSC-suppressing effect of metformin, and thereby overcome metformin resistance.

## Materials and Methods

### Cell lines and culture conditions

Eight human CRC cell lines (HT29, DLD-1, LoVo, HCT116, SW620, WiDr, COLO205, and Caco2) were used to evaluate the suppressive effect of metformin on CSCs of CRC. Metformin-sensitive HT29 cells and metformin-resistant SW620 cells were used in the experiments to elucidate the detailed mechanisms of the differential effects of metformin on CSCs. Eight human CRC cell lines were purchased from ATCC (Manassas, VA) and the Korean Cell Line Bank (Seoul, Republic of Korea). The cells were maintained in Dulbecco’s modified Eagle’s medium (DMEM) with 10% fetal bovine serum (FBS) (HyClone, Logan, UT) and 1% penicillin/streptomycin (Invitrogen, Carlsbad, CA). The cells were incubated in a 5% CO_2_ chamber at 37 °C.

### Drugs, antibodies, and RNAi

Metformin was purchased from Sigma (St. Louis, MO). Compound 968 (glutaminase C inhibitor), AICAR (AMPK activator), A769662 (direct AMPK activator), and rapamycin (mTOR inhibitor) were purchased from Merck Millipore (Darmstadt, Germany), Cell Signaling Technology (Danvers, MA), and Santacruz (Delaware, CA), respectively. Sodium oxamate was purchased from Sigma (St. Louis, MO). The antibodies used for flow cytometry, western blotting, and immunohistochemical (IHC) analysis were: anti-AMPK, anti-pAMPK((Thr172), anti-S6, anti-pS6(Ser235/236), anti-ASCT2, anti-PROM1 (CD133) (Cell Signaling Technology, Danvers, MA), anti-CD44, anti-c-Myc (Santacruz, Delaware, CA), anti-GLS1 (Abcam, Massachusetts, US), phycoerythrin (PE)-conjugated anti-PROM1 (CD133), and fluorescein (FITC)-conjugated anti-CD44 (BD Biosciences, Franklin Lakes, NJ).

For small interfering RNA (siRNA) experiments, HT29 and SW620 cells were transfected with a pool of 2~4 siRNAs against c-MYC, GLS1 and ASCT2. siRNAs were transfected using jetPRIME (Polyplus, New York, NY) with pools of siRNAs as indicated with a final siRNA concentration of 20 nM. Cells were transfected twice, first by reverse transfection and 24 hr later by forward transfection as outlined in the manufacturer’s instructions. Sequences are as follow: siRNA c-MYC #1 CAUCAUCAUCCAGGACUGUAUTT, siRNA c-MYC #2 CGAGCUAAAACGGAGCUUUTT, siRNA GLS1 #1 CCUGAAGCAGUUCGAAAUA, siRNA-GLS1 #2CUGAAUAUGUGCAUCGAUA, siRNA-GLS1 #3 AGAAAGUGGAGAUCGAAAU, siRNA-GLS1 #4 GCACAGACAUGGUUGGUAU, siRNA-ASCT2 #1 CCGGUCCUGUACCGUCCUCAA, siRNA-ASCT2 #2 UCGCUCAUACUCUACCACCUA.

### Flow-cytometric analysis and fluorescence-activated cell sorting (FACS)

CRC cells were plated at a density of 2 × 10^5^ cells/well in six-well plates in 2 ml medium, treated with metformin and other reagents, and incubated in a 5% CO_2_ chamber at 37 °C. After 48 h, flow-cytometric analysis of CSC markers (CD133 and CD44) was performed. The prepared cells were detached by accutase (Millipore, Billerica, MA) and resuspended in FACS buffer (1 × PBS, 1% bovine serum albumin and 2 mM ethylene diamine tetra-acetic acid). Primary antibodies (PE-conjugated anti-CD133 and FITC-conjugated anti-CD44) were added and incubated for 10 min at 4 °C. Then, the samples were washed with FACS buffer and analyzed using a FACSVerse (BD Biosciences, San Diego, CA) coupled to a computer with BD FACSuite software for data analysis. For sorting CSCs and non-CSCs, FTIC-CD44^+^/PE-CD133^+^ double positive cells and FITC-CD44^−^/PE-CD133^−^ double negative cells from HT29 and SW620 cells were sorted by using AriaIII flow cytometer(Becton Dickinson, San Jose, CA, USA).

### Tumor sphere-culture assay

To evaluate the sphere-forming abilities of the CSCs, a tumor sphere-culture assay was performed. SW620 and HT29 cells were plated at a density of 1,000 cells/well in 24-well ultra-low adhesive plates (Corning Incorporated, NY) in 1 ml serum-free medium with metformin (5 mM, 10 mM, or 20 mM). The serum-free medium consisted of DMEM-F12 supplemented with B27 (Life Technologies, Carlsbad, CA), 20 ng/ml epidermal growth factor (EGF), 10 ng/ml basic fibroblast growth factor (R&D Systems, Minneapolis, MN), 1% penicillin/streptomycin, and 2 mM L-glutamine (Life Technologies, Carlsbad, CA). The same medium without L-glutamine was also used to estimate the sphere-forming abilities of the CSCs in a glutamine-free state. The cells were incubated in a 5% CO_2_ chamber at 37 °C, and 500 μL of the culture medium was changed every 48 h. After 14 days, the number of tumor spheres was counted under a microscope (Olympus Bx51 microscope).

### Western-blot analysis

To analyze the AMPK-mTOR pathway, the expression of phosphorylated AMPK (pAMPK) and phosphorylated S6 (pS6) after metformin treatment was evaluated. In addition, the expression of glutaminase 1 (GLS1), and glutamine transporter (ASCT2) was assessed. The prepared cells were lysed using a protein extraction solution (iNtRON Biotechnology, Gyeonggi, Republic of Korea). After protein quantification, 10 μg protein extracts was fractionated using 10% or 15% sodium dodecyl sulfate polyacrylamide gel electrophoresis and then transferred to a polyvinylidene fluoride membrane (Bio-Rad, Hercules, CA). After blocking with 5% skim milk, the membrane was incubated with primary antibodies overnight at 4 °C. Subsequently, the membrane was incubated with secondary antibodies for 2 h at room temperature. To express the light emission of the proteins, an ECL (enhanced chemiluminescence) western blotting detection kit (Amersham Biosciences, Freiburg, Germany) was used, and the expressed light was captured on Kodak image film.

### Cellular respiration assay

We assessed cellular respiration in SW620 and HT29 cells using an XF (extracellular flux) analyzer (Seahorse Bioscience, North Billerica, MA). The XF analyzer simultaneously measures the oxygen consumption rate (OCR), as an indicator of OXPHOS, and the extracellular acidification rate (ECAR), as an indicator of glycolysis. The prepared cells were washed in XF assay medium and maintained in 225 μL/well XF medium in a non-CO_2_ incubator for 1 h at 37 °C. During the incubation of the cells, DMSO in XF assay media was loaded into injection port A of each well in the XF96 sensor cartridge. The XF assay protocol was started by an initial calibration of the cartridge in the XF analyzer followed by the measurement of the OCR and ECAR from the cell plate. Oligomycin, FCCP (p-trifluoromethoxyphenylhydrazone), and rotenone were added sequentially. Oligomycin inhibits the complex V in the mitochondria and decreases the OCR. FCCP targets the inner mitochondrial membrane and increases the OCR. Rotenone inhibits the complex I in the mitochondria and decreases the OCR.

### Glutamate assay

To measure glutamate, HT29 and SW620 cells were plated at a density of 2 × 10^5^ cells/well in six-well plates, treated with metformin and compound 968, and incubated for 48 h. The amount of glutamate in these cell lysates were determined by an enzymatic reaction using a glutamate assay kit (Biovision, Milpitas, CA) according to the manufacturer’s protocol. Enzymatic reactions were measured by spectrophotometry at 450 nm.

### *In vivo* mouse xenograft experiments

Six-week-old male BALB/c athymic nude mice were purchased from Orient Bio (Gyeonggi, Republic of Korea) and acclimated for 1 week. This study was approved by the Committee of Care and Use of Laboratory Animals of Yonsei University College of Medicine. All mouse experiments were performed according to institutional guidelines and policies.

SW620 cells or HT29 cells were suspended in Matrigel (BD Bioscience) at a concentration of 1 × 10^6^ cells/200 μl, diluted 1:1 in DMEM, and subcutaneously injected into both flanks of the mice. After 1 week, the implanted mice were randomly divided into four groups (5 mice per group): control, metformin (metformin only), 968 (compound 968 only), and combined (metformin and compound 968). The metformin was dissolved in water and supplied to the mice through drinking water (500 mg/kg/day). Compound 968 was injected intraperitoneally (10 mg/kg with 10% DMSO in 200 μl PBS) on a daily basis. Vehicle control (10% DMSO in 200 μl of PBS) was injected intraperitoneally into the mice of the control and metformin groups. Tumor sizes were measured every other day using calipers, and tumor volumes were calculated based on the following formula: tumor volume = length × [width]^2^/2. All mice were sacrificed at 17 days after the first drug treatment, and the tumor masses were dissected. The dissected tumors were placed in 10% buffered formalin for immunohistochemistry (IHC).

### Immunohistochemistry for CD133 and CD44

IHC was performed on frozen tumor sections for CD133 and on 4 μm sections of formalin-fixed, paraffin-embedded, dissected tumor samples for CD44. The paraffin-embedded sections were deparaffinized in xylene and rehydrated in gradually decreasing concentrations of ethanol. Antigen retrieval was performed using sodium citrate buffer (10 mM, pH 6.0) in a heated pressure cooker for 5 or 7 min. After incubation with 3% hydrogen peroxide to block endogenous peroxidase activity for 30 min, the sections were incubated in a blocking reagent for 30 min at room temperature. Anti-CD133 antibody (1:100 dilution) or anti-CD44 antibody (1:100 dilution) was incubated with the sections overnight at 4 °C, and then secondary antibody was incubated for 30 min at room temperature. After slides were developed with a Vectastain ABC kit (Vector Laboratories), immunostaining was performed using DAB solution (Dako, Carpinteria, CA). After counterstaining with hematoxylin, IHC staining was evaluated by light microscopy, and immunoactivity was assessed based on the proportion of immunostained tumor cells. After IHC staining, a researcher (JH Kwon) randomly assigned new identification codes to each slide for a blind test and provided the slides to two researchers (JH Kim and KJ Lee) who then independently interpreted the staining. The CD133-stained or CD44-stained cells among 100 tumor cells were counted in five different fields (total = 500 counted cells) with 400× magnification, and the cells counts were then converted into percentages.

### Tumor organoid culture of human CRC tissues and drug treatment

For tumor organoid culture from CRC patients, this study was approved by the Institutional Review Board of Yonsei University College of Medicine, Severance Hospital (IRB No. 4-2012-0859), and endoscopic biopsy samples were obtained from CRC patients with informed consent (Supplementary Table 1). All research with patients’ tumor organoids was performed in accordance with relevant guidelines and regulations.

The biopsy samples were washed in PBS with 100 μg/ml primocin (InvivoGen, San Diego, CA) and chopped into approximately 0.5 mm pieces. Next, the tissues were incubated in digestion buffer (DMEM, 2.5% FBS, 6.25 mg/ml collagenase type IX [Sigma, St. Louis, MO]) for 30 min at 37 °C. After the digestion, the isolated tumor cells were washed in PBS and embedded in Matrigel on ice (growth-factor reduced, phenol red free; BD Bioscience) and then seeded on 48-well plates (500 crypts/25 μL Matrigel per well). The Matrigel was polymerized for 15 min at 37 °C and then overlaid by 250 μL/well basal culture medium (advanced DMEM/F12 with 1% penicillin/streptomycin, Glutamax, 1 × N2, 1 × B27 without retinoic acid [all from Invitrogen], 2 mM L-glutamine [Life Technologies], 50 ng/ml EGF [R&D Systems], 1 mM N-Acetyl-L-cysteine, 10 mM Nicotinamide, 10 nM Gastrin I, 10 μM SB202190, 500 nM A-83-01, 2.5 μM PGE2, 1 μg/ml R-spondin 1, 100 ng/ml Noggin, and 10 μM Y27632 [all from Sigma]).

The culture medium was changed every 2 days with fresh basal culture medium. For passage, organoids and Matrigel were washed in 1 mL PBS/well, mechanically disrupted using a 1000 μl pipette, and dissociated with trypsin/EDTA (Sigma). The dissociated organoids were embedded in Matrigel and seeded in 48-well plates (500 crypts/25 μL Matrigel per well). The Matrigel was polymerized for 15 min at 37 °C and then overlaid with 250 μL/well basal culture medium with metformin (2 mM or 4 mM), compound 968 (10 μM), or metformin (2 mM) and compound 968 (10 μM). The culture medium was changed every 2 days.

### Statistical analysis

Statistical analysis was performed using IBM SPSS Statistics version 20.0 (IBM Co., Armonk, NY, USA). Student’s t-tests and chi-square tests were performed for continuous and categorical variables, as appropriate. *P* values lower than 0.05 were considered statistically significant.

## Electronic supplementary material

Supplementary Information
