# Publisher Correction: Effects of metformin on colorectal cancer stem cells depend on alterations in glutamine metabolism

**DOI:** 10.1038/s41598-018-29895-5

**Published:** 2018-08-28

**Authors:** Jae Hyun Kim, Kyoung Jin Lee, Yoojeong Seo, Ji-Hee Kwon, Jae Pil Yoon, Jo Yeon Kang, Hyun Jung Lee, Soo Jung Park, Sung Pil Hong, Jae Hee Cheon, Won Ho Kim, Tae Il Kim

**Affiliations:** 1 Department of Internal Medicine, Yonsei University College of Medicine, Seoul, Korea; 2 Institute of Gastroenterology, Yonsei University College of Medicine, Seoul, Korea; 3 Cancer Prevention Center, Yonsei University College of Medicine, Seoul, Korea; 4 Brain Korea 21 PLUS Project for Medical Science, Yonsei University, Seoul, Korea

Correction to: *Scientific Reports* 10.1038/s41598-017-18762-4, published online 11 January 2018

The original version of this Article contained a typographical error in the spelling of the author Tae Il Kim, which was incorrectly given as Tae II Kim. This has now been corrected in the PDF and HTML versions of the Article.

