# Supplementary material for: Effects of metformin on colorectal cancer stem cells depend on alterations in glutamine metabolism
Source: Sci Rep. 2018 Jan 11;8:409. doi: 10.1038/s41598-017-18762-4 (PMC5765079; doi:10.1038/s41598-017-18762-4)
Supplement: Supplementary file 1 — Supplementary Information [file 41598_2017_18762_MOESM1_ESM.doc]

**Effects of metformin on colorectal cancer stem cells depend on alterations in glutamine metabolism**

Jae Hyun Kim^1,2^, Kyoung Jin Lee^2^, Yoojeong Seo^2^, Ji-Hee Kwon^2^, Jae Pil Yoon^2^, Jo Yeon Kang^2^, Hyun Jung Lee^1,2^, Soo Jung Park^1,2^, Sung Pil Hong^1,2^, Jae Hee Cheon^1,2^, Won Ho Kim^1,2^ and Tae Il Kim^1,2,3 *^

^1^Department of Internal Medicine, ^2^Institute of Gastroenterology, ^3^Cancer Prevention Center, Yonsei University College of Medicine, Seoul, Korea

Running title: Effect of metformin and glutamine on colorectal cancer stem cells

***Corresponding Author**: Tae Il Kim, MD, PhD,

Department of Internal Medicine, Institute of Gastroenterology, Cancer Prevention Center,

Yonsei University College of Medicine,

50-1 Yonsei-ro, Seodaemun-gu, Seoul 120-752, Korea

Tel: +82-2-2228-1965, e-mail: taeilkim@yuhs.ac

**Figure S1.** Expression of c-Myc, GLS1 and ASCT2 in colorectal cancer cell lines.

**Figure S2.** Effect of metformin and/or compound 968 on cancer stem cells of colorectal cancer cell lines.

**Figure S3.** Effect of metformin and/or LDH-A inhibitor on cancer stem cells of SW620 and HT29 cells.

**Figure S4.** The original blot images of the cropped images in Fig. 4c.

**Table S1.** The summarized information of two patients with colorectal cancer who were examined for tumor organoid culture.


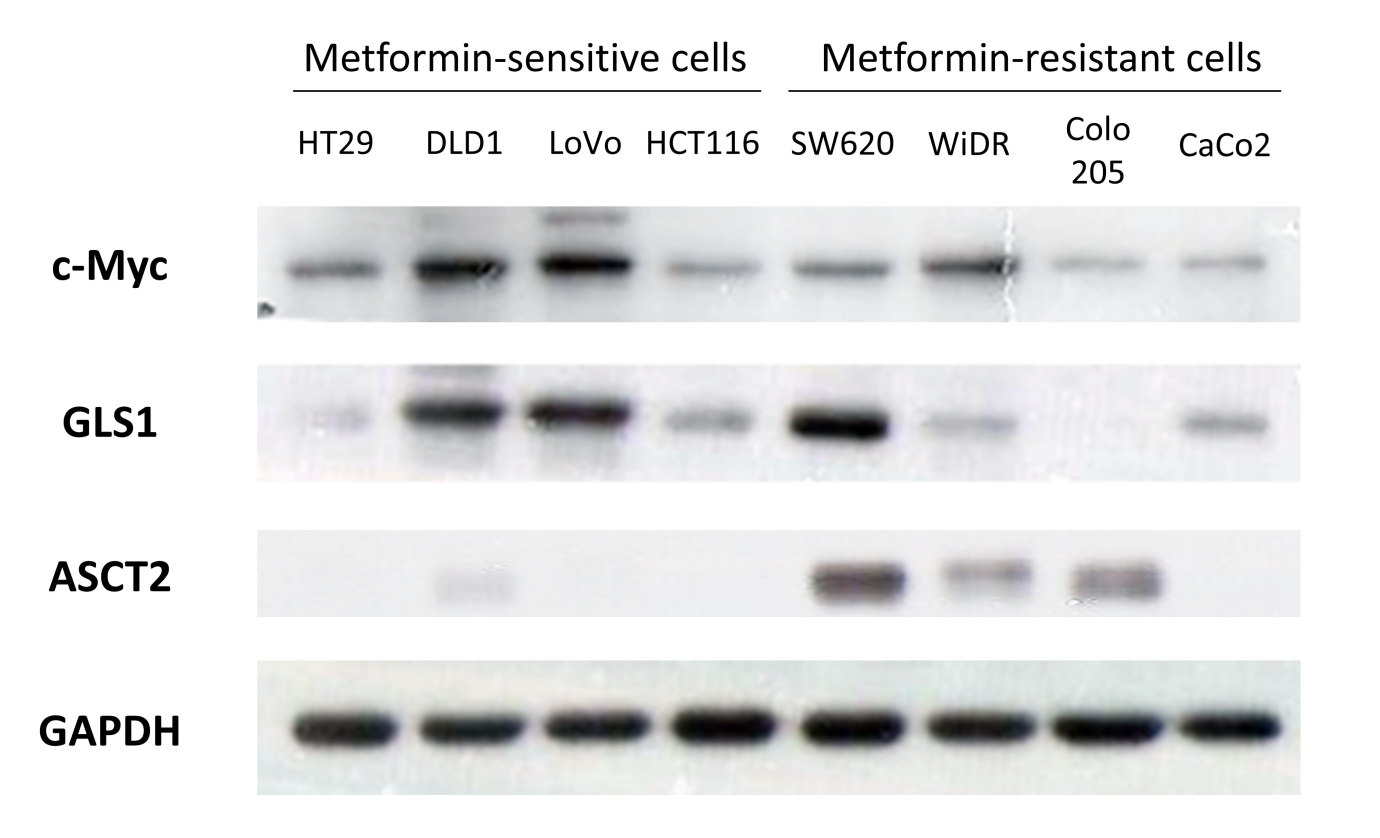


**Supplementary Figure S1.** Expression of c-Myc, GLS1 and ASCT2 in colorectal cancer cell lines. Protein expression of c-Myc, GLS1 and ASCT2 in metformin-sensitive cells (HT29, DLD1, LoVo and HCT116) and metformin-resistant cells (SW620, WiDR, Colo205 and CaCo2) were evaluated by Western-blot analysis.


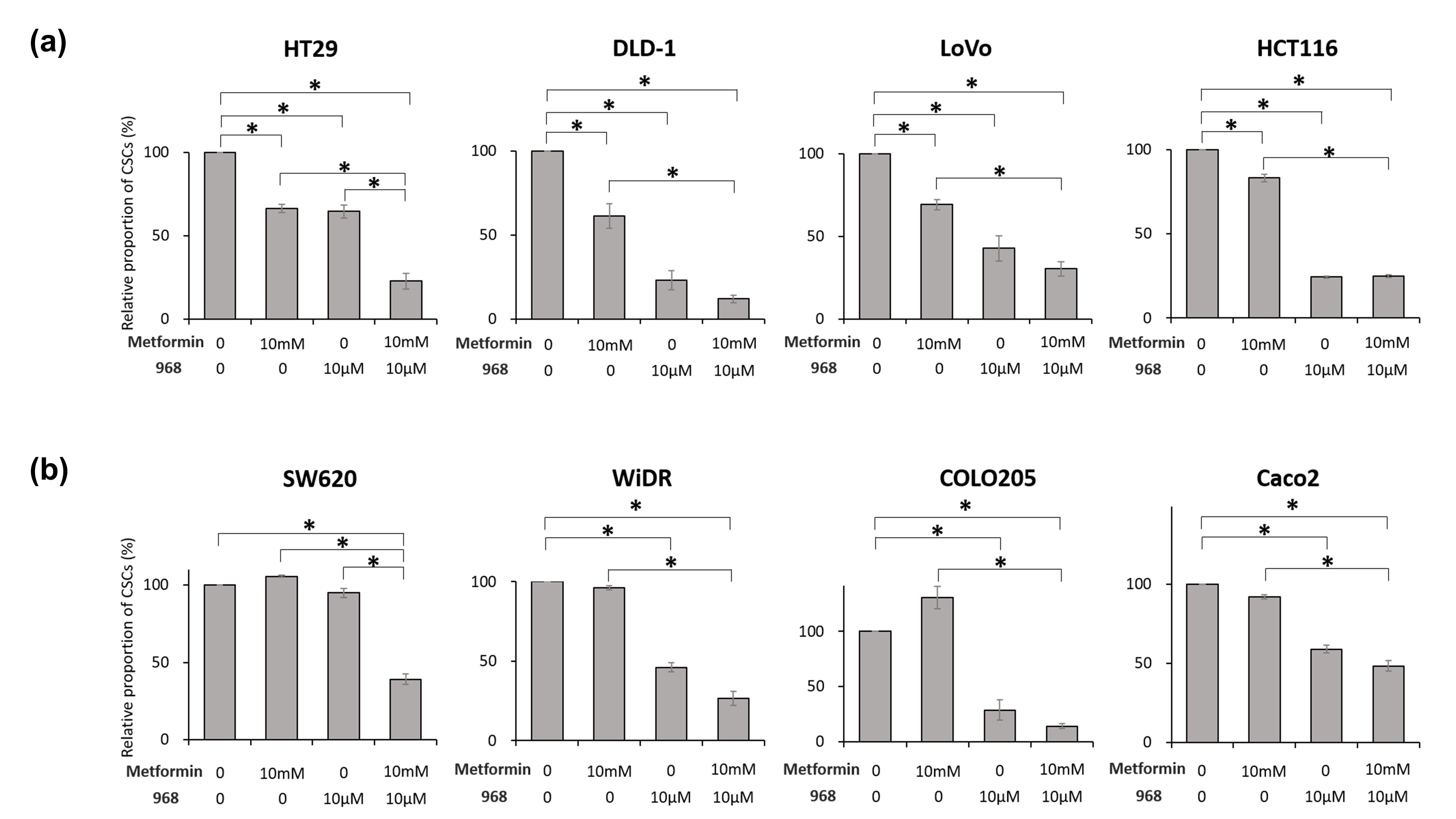


**Supplementary Figure S2.** Effect of metformin and/or compound 968 on cancer stem cells of colorectal cancer cell lines. In metformin-sensitive cell lines (HT29, DLD-1, LoVo, and HCT116) (a) and metformin-resistant cell lines (SW620, WiDR, COLO205, and Caco2) (b), the proportion of CD133^+^CD44^+^ cells was analyzed by flow cytometry after 48 h treatment with control vehicle, metformin (10 mM), compound 968 (10 μM), or a combination of metformin (10 mM) and compound 968 (10 μM). Data are expressed as the mean ± standard error of three independent experiments; * *P* < 0.05 (compared with control).


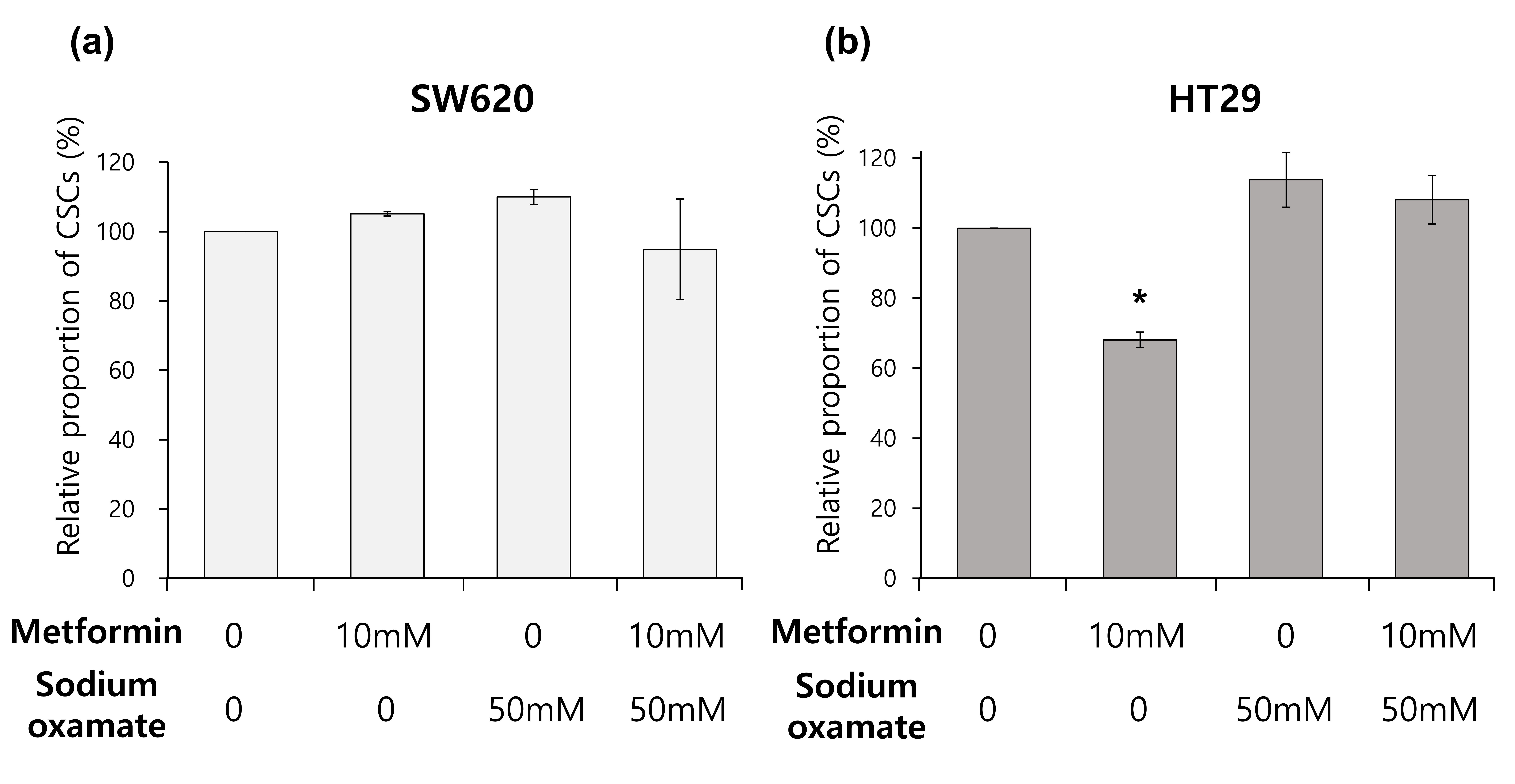


**Supplementary Figure S3.** Effect of metformin and/or LDH-A inhibitor on cancer stem cells (CSCs) of SW620 and HT29 cells. (a and b) Inhibition of LDH-A with or without metformin showed no suppressing effect on CSCs of SW620 and HT29 cells. The proportion of CSCs were analyzed by flow cytometry after 48-hr treatment with control, 10mM metformin, 50 mM sodium oxamate, or a combination of 10 mM metformin and 50 mM sodium oxamate. Data are expressed as the mean ± standard error of three independent experiments; * *P* < 0.05 (compared with control).

**Supplementary Figure S4.** The original blot images of the cropped images in Fig. 4c.

In Western-blot analysis, the expression of GLS1 and ASCT2 in SW620 and HT29 cells was analyzed after 48 h treatment with control vehicle or metformin (5 mM, 10 mM, or 20 mM). CSCs (CD133^+^CD44^+^) and non-CSCs (CD133^-^CD44^-^) were sorted using FACS after the same treatment.

**Supplementary Table S1.** The summarized information of two patients with colorectal cancer who were examined for tumor organoid culture.

|  | **Gender** | **Age** | **Location** | **Differentiation** | **EGFR** | **KRAS** | **MSI status** |
| --- | --- | --- | --- | --- | --- | --- | --- |
| **Patient a** | Female | 54 | Rectum | Moderate | Weak positive | Wild | MSS |
| **Patient b** | Male | 74 | Rectum | Moderate | Weak positive | Mutation | MSI-Low |

MSI, microsatellite instability; MSS, microsatellite stable
